# Inferring the impact of humanitarian responses on population mortality: methodological problems and proposals

**DOI:** 10.1186/s13031-023-00516-x

**Published:** 2023-03-30

**Authors:** Francesco Checchi

**Affiliations:** Department of Infectious Disease Epidemiology, Faculty of Epidemiology and Population Health, London School of Hygiene and Tropical Medicine, London, UK

**Keywords:** Humanitarian assistance, Humanitarian response, Crisis, Emergency, Mortality, Death rate, Attribution, Causality, Impact, Effect, Methods

## Abstract

Reducing excess population mortality caused by crises due to armed conflict and natural disasters is an existential aim of humanitarian assistance, but the extent to which these deaths are averted in different humanitarian responses is mostly unknown. This information gap arguably weakens governance and accountability. This paper considers methodological challenges involved in making inferences about humanitarian assistance’s effect on excess mortality, and outlines proposed approaches. Three possible measurement questions, each of which contributes some inferential evidence, are presented: (1) whether mortality has remained within an acceptable range during the crisis (for which different direct estimation options are presented); (2) whether the humanitarian response is sufficiently appropriate and performant to avert excess mortality (a type of contribution analysis requiring in-depth audits of the design of humanitarian services and of their actual availability, coverage and quality); and (3) the actual extent to which humanitarian assistance has reduced excess deaths (potentially the most complex question to answer, requiring application of causal thinking and careful specification of the exposure, and for which either quasi-experimental statistical modelling approaches or a combination of verbal and social autopsy methods are proposed). The paper concludes by considering possible ‘packages’ of the above methods that could be implemented at different stages of a humanitarian response, and calls for investment in improved methods and actual measurement.

## Background

### Humanitarian assistance and mortality reduction

Humanitarian assistance (HA) has an existential aim of minimising population mortality directly or indirectly attributable to crisis conditions due to armed conflict, natural disasters and other destabilising events. The latter quantity may be thought of as ‘excess’ deaths that would have not occurred in a counterfactual no-crisis scenario [1, 2]. More specifically, it may be argued that HA should aim to mitigate the crisis’ impacts, not pre-existing conditions, however dire: accordingly, an appropriate metric for the success of HA could be its effect on *excess* rather than overall mortality, though reducing the latter may be a desirable and pragmatic secondary aim. Furthermore, the effectiveness of HA should only be judged against crisis-attributable mortality that it could have reasonably mitigated: for example, in Syria many deaths due to indiscriminate use of weapons or deliberate attacks against civilians cannot reasonably be ascribed to a failure of HA. On the other hand, humanitarian action writ large also includes advocacy for and support to civilian protection as well as promotion of respect for the laws of war by combatants, as exemplified by the work of the International Committee of the Red Cross: as such, its potential impact could also be viewed as encompassing deaths resulting from insufficient civilian protection and/or war crimes.

The current global HA system effectively substitutes, partially or fully, local government services. Humanitarian actors thus wield substantial power, and their decisions on resource allocation, design of humanitarian services, and their operational delivery, bear potentially large impacts on human survival. However, the humanitarian system’s governance, consisting of largely voluntary coordination mechanisms and unsystematic accountability processes, is criticised as being inadequate [3–6]. Moreover, crisis-affected people often have limited influence on HA and agency to challenge its authority; to some extent, this is also true for citizens of countries that contribute to HA worldwide.

Given the above, this paper premises that no arrangements for humanitarian governance and accountability can be considered sufficient unless they also include systematic efforts to determine the extent to which HA has fulfilled its existential mortality reduction aim. However, measurement of population mortality during humanitarian responses is inconsistent and rarely done at crisis-wide scale, and most crises do not occur in settings with functional demographic surveillance [7]. Moreover, HA inevitably occurs within a dynamic context in which other factors affecting mortality, such as insecurity and displacement, are also at play: disentangling from these the specific effect of HA is thus intrinsically complex. This paper considers options and methods for generating robust inferences on the mortality impact of HA.

### Specifying the question

While the broad question of whether HA reduces mortality is clear, it is useful to consider what, in practice, may be worth measuring. At least three qualitatively distinct questions, presented in Table 1, may be thought of as contributing *some* useful information. Firstly, if we can form a reasonable assumption of what an acceptable level of mortality may be (e.g. the range of death rate observed in the years prior to the crisis), we may content ourselves with ascertaining whether mortality has indeed remained within this range during the humanitarian response period. This question does not reveal how much HA has contributed to maintaining death rates below unacceptable levels, but it at least provides a crude ‘job done’ answer, or, alternatively, a measure of the extent of non-averted excess mortality.

**Table 1:** Overview of possible questions for measurement of the effect of humanitarian assistance on mortality

Question	Advantages	Limitations	Requirements for primary data collection	Expertise required
Has mortality remained within an acceptable range Throughout the crisis? Throughout the period during which HA was available?	Pragmatic	No evidence is generated that HA has actually helped and has been efficient No information is generated on specific strengths/weaknesses of the humanitarian response	Moderate	Low
To what extent has the humanitarian response put in place services that, given the context, are plausibly sufficient to minimise excess mortality?	Emphasises specific strengths and weaknesses of the response: the evidence generated may have immediate utility and enhance governance	Less compelling to non-technical decision-makers than the other questions Difficult to compare results across crises	Moderate to high (depends on functionality of humanitarian information systems)	Low
By how much has HA averted excess mortality?	Compelling evidence for non-technical decision-makers Estimates can be combined with other data to quantify efficiency	Results will likely come too late for action No information on specific strengths/weaknesses of the humanitarian response will be generated	High	High

A second possible line of inquiry is to set aside actual measurement of mortality, and focus instead on ascertaining the extent to which the humanitarian response has matched the potential for excess mortality with appropriate and sufficiently performant services targeting the factors driving excess deaths—for example, whether crisis-resultant food insecurity has been tackled; poor sanitation ameliorated through effective water, sanitation and hygiene interventions; or vaccines used appropriately to reduce the risk of endemic and epidemic diseases. Answering this question will not provide direct evidence on mortality effects, but should generate actionable information on specific gaps and weaknesses in the response.

Lastly, a direct question may be asked of how much excess mortality has indeed been averted by HA: providing an answer to this question implies that causality has been established with reasonable confidence. Such an analysis may also be extended to explore questions of efficiency and equitable resource allocation: for example, if it is estimated that HA averted N deaths, a crude estimate of the cost per death averted may also be computed, potentially enabling comparisons across different crises.

While all three questions yield useful information, methods required to answer them may be varyingly resource-intensive and feasible. Realistic methodological options to answer each of the questions are thus explored below.

## Methodological options

### Question 1: Has mortality remained within an acceptable range?

#### What range is acceptable?

In 2017, hurricane Maria caused large-scale damage to the island of Puerto Rico. An initially low official estimate of the disaster-attributable death toll was later rectified by several scientific studies [8, 9] that compared pre- and post-hurricane vital registration statistics: in this example, an ‘acceptable’ level of mortality was readily available and comparable with statistics after the sudden disaster. In most contemporary crises, however, establishing the counterfactual (what the death rate would have been in the absence of the crisis) is complicated by one or more of the following problems: (i) a robust, recent estimate of the pre-crisis death rate is not available, e.g. because the last census was conducted decades earlier and/or the crisis is extremely protracted, as in the Democratic Republic of Congo, Somalia and Afghanistan; (ii) a specific, acute emergency may occur in a setting already affected by crisis, as in the example of drought and food insecurity in Somalia, where insecurity has lasted three decades: the specific, additional emergency responses launched following droughts in 2010 and 2016 aimed to reduce drought-related mortality specifically [10, 11], not the pre-existing death rate; (iii) while a reasonable countrywide estimate may be assumed from demographic models, within-country disparities may be substantial, i.e. countrywide levels may not reflect baseline conditions in the crisis-affected region; and (iv) as crises become protracted, assuming fixed counterfactuals becomes questionable: it is plausible that, in the absence of a crisis, a secular decline in mortality levels would have occurred due to factors such as changing age distribution, reduced fertility, improved livelihoods and better services.

**Fig. 1:**
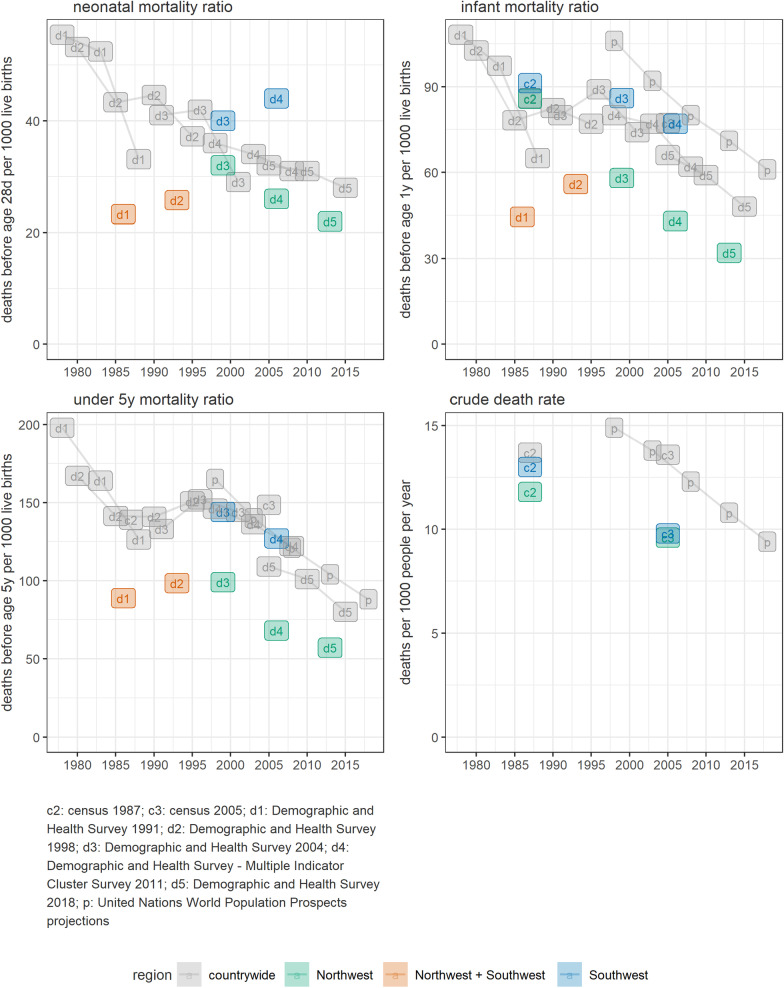
Trends in selected mortality indicators, Cameroon. Data points are positioned on the x axis at the midpoint of the period that the estimates cover. Estimates come from Demographic and Health Surveys [12–16], national census reports [17] or United Nations World Population Prospects [18]

As an example of the latter two challenges, since late 2017, the Northwest and Southwest Regions of Cameroon have experienced intense insecurity and displacement. Fig. 1 shows available estimates of mortality in children and all ages in these regions and Cameroon as a whole: these indicate that at least the Northwest had a lower pre-crisis baseline than countrywide, and that a secular declining trend would plausibly have continued in the crisis-affected regions, had there been no conflict, i.e. that the ‘acceptable’ mortality threshold should be revised downwards year-on-year. Generally, the choice of an acceptable range (by age group) should be conservative, locally specific and supported by demographers familiar with the context, particularly where missing or contradictory pre-crisis estimates necessitate careful assumptions and use of other proxy variables.

#### Which sources of data?

While the obvious source of mortality data anywhere should be vital events registration systems, in practice these systems are absent or insufficiently functional apart from rare exceptions (e.g. the occupied Palestinian Territories, Ukraine). Methods to fill this information gap with population-representative estimates of key mortality indicators, including the crude and under 5 years death rate, are reviewed extensively elsewhere [19]. Briefly, these may be divided into four broad approaches.

Firstly, prospective demographic surveillance may be established either exhaustively or in sentinel sites: at its simplest, this consists of home visitors collecting weekly or monthly data on births, deaths and migration from households [20]. If conceived as a de novo, top-down system, such systems may be relatively burdensome and, without sustained support, decay over time [21]. They do, however, furnish actionable real-time information [22]. In certain scenarios, monitoring of burial sites, either on-site or remotely through satellite imagery [23], may replace household-based data collection.

Secondly, retrospective surveys are an option to estimate death rates by interviewing a sample of households about the composition and evolution (deaths, births, in- and out-migration) during a given ‘recall’ period [24]; ‘humanitarian’ mortality surveys are considerably simpler than in-depth demographic studies (e.g. Demographic and Health Surveys), and their methods and analysis are highly standardised through the Standardised Monitoring of Relief and Transitions (SMART) initiative [25]. SMART surveys are primarily done to measure the prevalence of acute malnutrition, but usually contain a mortality module; they usually limit their sampling frame to an administrative level 2 unit (e.g. district) or single camp, but larger-scale surveys have been conducted [26–29]. The main limitations of surveys are that they report on the past and don’t generate actionable information on the drivers of mortality. Surveys are also highly prone to both sampling and response (i.e. information) biases if conducted with insufficient expertise, training and community engagement [30].

Thirdly, interviews of carefully selected community key informants may be combined with any existing ‘lists’ of recent decedents and statistical capture-recapture analysis to estimate the true death toll [31, 32]. This approach has been used seldom, but is economical [33], relies on community resources and, once established locally, could be implemented regularly to provide frequent mortality updates. We have written openly available scripts for analysing such informant data (see https://github.com/francescochecchi/mortality_capture_recapture_analysis).

Lastly, we have recently used small-area estimation principles to develop statistical models, built from a wealth of routinely collected data on variables on the causal pathway to mortality and validated on available SMART surveys, that retrospectively estimate crude death rates with reasonable accuracy in Somalia [34], South Sudan [35] and north east Nigeria [36]. This method is very burdensome as it involves extensive negotiation for data access, data curation and statistical analysis, in addition to a separate task of reconstructing population denominators to account for internal and refugee displacement [37]. We are currently exploring its potential for forecasting, which could offer an alternative to primary data collection.

Whichever method is locally preferable, the frequency of data collection would depend on whether question 1 is being asked to inform real-time response, or merely during retrospective evaluation; the former is clearly preferable. Moreover, while little empirical evidence exists on the robustness of household recall of past demographic events, recall periods longer than a year may entail increasing information bias [30], suggesting the need for at least yearly surveys. Similarly, some geographic granularity in estimation is recommendable (as opposed to a single estimate for the entire crisis-affected population), so as to identify sub-regions requiring strengthened support and highlight inequities in the response: this entails independent samples for each geographic unit of interest, and thus an expansion in sample size.

### Question 2: Have humanitarian services addressed avertable excess mortality causes?

#### What determines the effect of HA on mortality?

**Fig. 2:**
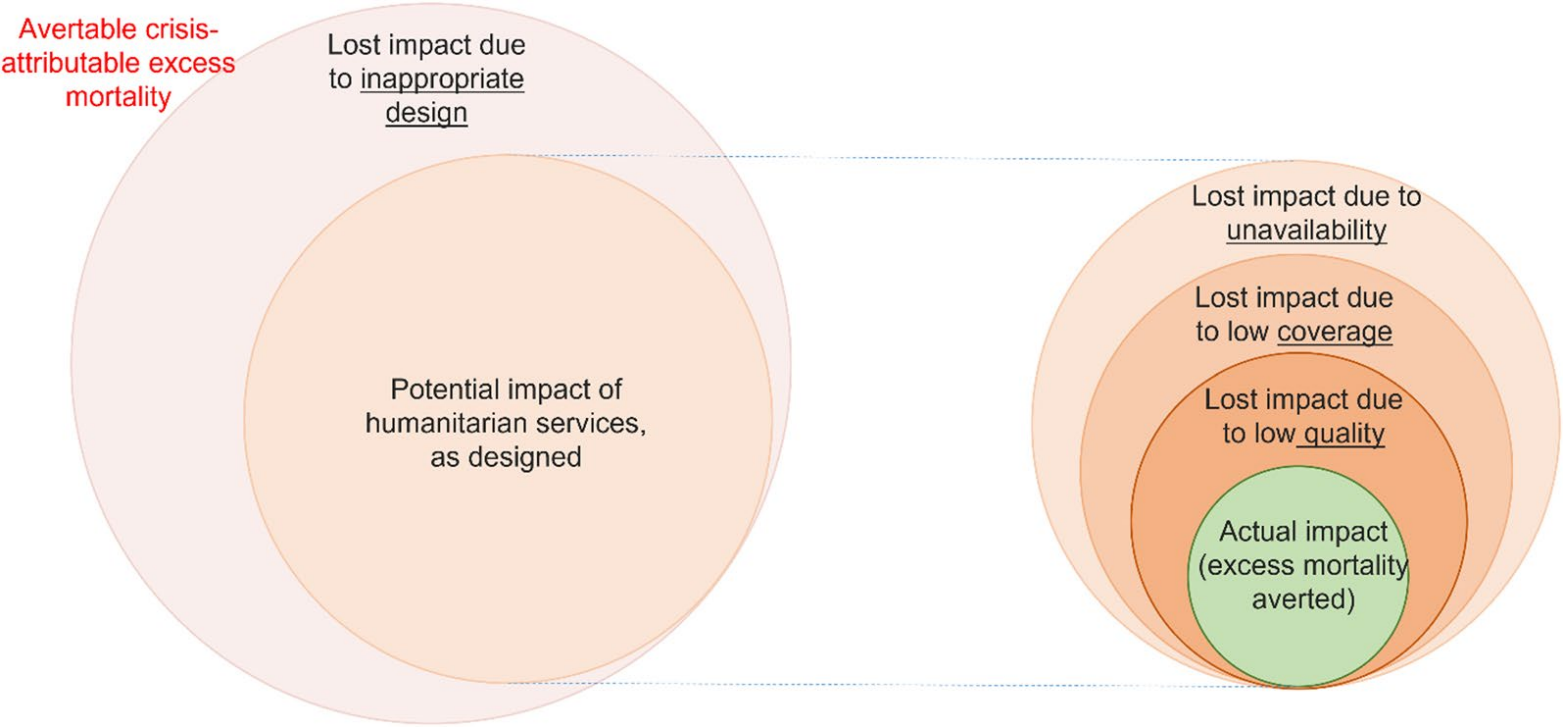
Graphical representation of mathematical determinants of the effect of humanitarian assistance on excess mortality

How exactly does HA reduce excess deaths? One way to represent this mechanism through potentially quantifiable, concretely meaningful parameters is, as depicted in Fig. 2, to consider that, for any given time horizon of interest, the crisis will result in a total amount of excess deaths that HA can theoretically avert. This potential impact will be diminished if the humanitarian response is inappropriate, i.e. designed and resourced in such a way that, even if implemented perfectly, it misses opportunities to avert a certain proportion of excess deaths. *Appropriateness* problems could be further classified into the rubrics of ‘what’ (e.g. a response omits mass vaccination against cholera despite a high epidemic threat), ‘how’ (outpatient health services overly rely on infrequent mobile clinics instead of accessible, fixed health posts) and ‘for whom’ (response efforts are concentrated in government-held areas and neglect people in opposition-held areas) [38]. Impact is further diminished due to imperfect *performance*, which is itself a function of availability (e.g. cash transfers for food-insecure households have been funded, but are held up by disorganisation), coverage or utilisation (the proportion of people in need of a service who actually access it: e.g. low coverage of facility-assisted births) and quality (whether services such as community management of acute malnutrition or water purification are delivered per good practice standards).

#### Tracking the logic model

**Fig. 3:**
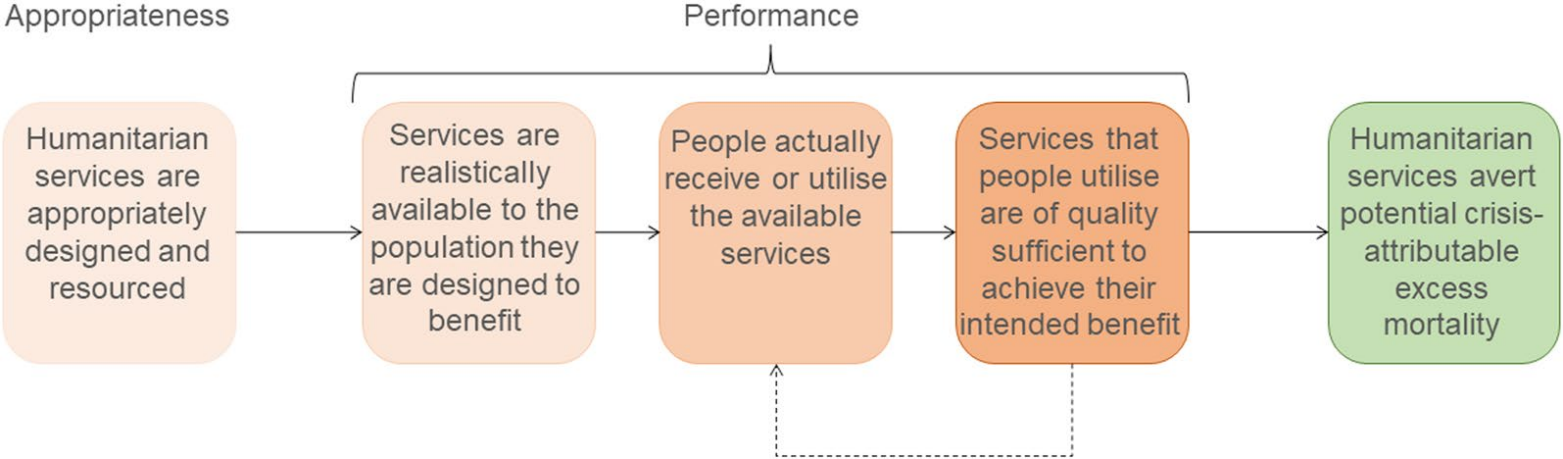
Logic model representing steps in the mechanism of reduction in excess mortality through humanitarian assistance. The dashed arrow denotes a feedback loop

The above arithmetic can be represented as a logic model (Fig. 3). Contribution analysis [39] may then be used to closely ‘audit’ each step in the model. For appropriateness, we are developing a software-assisted audit exercise that covers the health and nutrition sectors: it systematically considers the ‘what’ (package of services offered, versus likely burden of disease and epidemic threats and known gaps), ‘how’ (extent to which services are designed to support the health system and mitigate known access barriers) and ‘for whom’ (rationale for selecting the target population) questions. For performance, a menu of availability, coverage and quality indicators should be considered, and criteria for shortlisting these based on the package of services offered and ease of measurement. While these audits would mostly rely on existing documents and data, the latter (e.g. health facility utilisation statistics) would need to be available and reliable; it may be necessary to supplement these with rapid surveys of service coverage, increasing the burden of data collection for this approach. Lastly, the approach would require collaborative work by sectoral experts and information managers or evaluation scientists. Its summative output would be a semi-quantitative consensus statement of the extent to which the humanitarian response is indeed fit for its mortality reduction purpose. Usefully, these audit exercises would indicate problems and gaps in the response’s design or implementation, i.e. provide actionable information, particularly if conducted regularly in real-time.

### Question 3: By how much has humanitarian assistance reduced excess mortality?

#### Dealing with causal inference

As framed, question 3 implies that we are confident that HA is the cause of any estimated effects on mortality. In epidemiology, causal inferences are generally made through a combination of statistical techniques, fundamental knowledge and careful plausibility reasoning. Bradford Hill’s considerations for causality [40] provide useful prompts, and are applied in Table 2 towards the question at hand.

**Table 2:** Bradford Hill’s causality considerations as applied to the association between humanitarian assistance and excess mortality

Consideration	Application to the HA-mortality association	Relevance (this author’s suggestion)
Strength (effect size)	A large observed effect size indicates that it is relatively more likely that HA is indeed causing a decrease in mortality	− (The effect of HA will depend on the appropriateness and performance of HA, i.e. the intensity of exposure: a small effect size does not particularly suggest lack of causation, as it may simply reflect insufficient HA.)
Consistency (reproducibility)	A comparable effect of HA is observed in multiple crises of comparable typology that benefit from similar humanitarian responses	+ (In practice, variability in crisis and response characteristics, as well as evaluation data and methods, may preclude reproducibility of effects observed in one crisis across other crisis scenarios.)
Specificity	Both HA and mortality are measured and quantified in an unequivocal way, and not in generic terms	++ (Mainly relevant for the exposure: as discussed in the text, the metric by which HA is quantified should reflect specific parameters, e.g. appropriateness, coverage etc. that determine its theoretical effect on mortality.)
Temporality	The decrease in mortality occurs after the increase in HA	++
Biological gradient	A (negative) ‘dose–response’ relationship is observed between HA and mortality: the more appropriate and performant HA, the lower mortality	++
Plausibility	The observed effect is consistent with the body of knowledge on what is known about the mechanism whereby specific humanitarian services (e.g. measles vaccination) affect mortality, and more generally about causal factors for mortality. The observed effect is also consistent with what is known about the specific context of the crisis in question, how HA was implemented and what other factors may have affected mortality	++
Coherence	Published evidence, e.g. from trials, shows that the humanitarian services offered as part of the response are effective against mortality	+ (More applicable to specific and new health problems of unknown cause. Somewhat overlapping with plausibility.)
Experiment	If HA is withdrawn the observed effect disappears. Alternatively, HA is offered only to a randomly selected group, and an effect is observed among those receiving HA but not among those not receiving it	− (Unethical, unless a new, specific intervention is proposed, the effectiveness of which is insufficiently established.)
Analogy	A similar causal association has been observed in other (non-crisis) settings where vulnerable people have received a given package of services. This could, for example, be stable but low-income countries where a basic package of services is introduced at scale	+ (It might be challenging to identify analogous associations outside of humanitarian responses. This consideration would probably not add much beyond the list above.)

Plus ( +) and minus (−) signs indicate the extent to which each consideration is relevant in the author’s judgment (− = not relevant; +/++  = somewhat / highly relevant)

Not all of Bradford Hill’s considerations may be amenable to measurement or indeed relevant. Causal inference should also be predicated upon a careful, exhaustive a priori causal framework. Directed acyclic graphs (DAGs) are increasingly used to depict the causal pathway of interest as well as other variables or factors that may need to be factored in the analysis [41]: specifically, a DAG provides clarity on which potential confounders (variables that influence both the exposure and the outcome, and whose presence may result in a spurious effect) need to be adjusted for in the analysis. If data to quantify a given confounder cannot be collected, the DAG will at least support interpretation of findings, by indicating potential bias. While a DAG, like the more familiar theory of change model, charts pathways from cause to effect, the latter is focussed on achieving consensus on how a given programme or intervention will achieve its intended goal, and does not consider confounding or other factors that will also affect the target: as such, it is less suitable for epidemiological and statistical analysis.

**Fig. 4:**
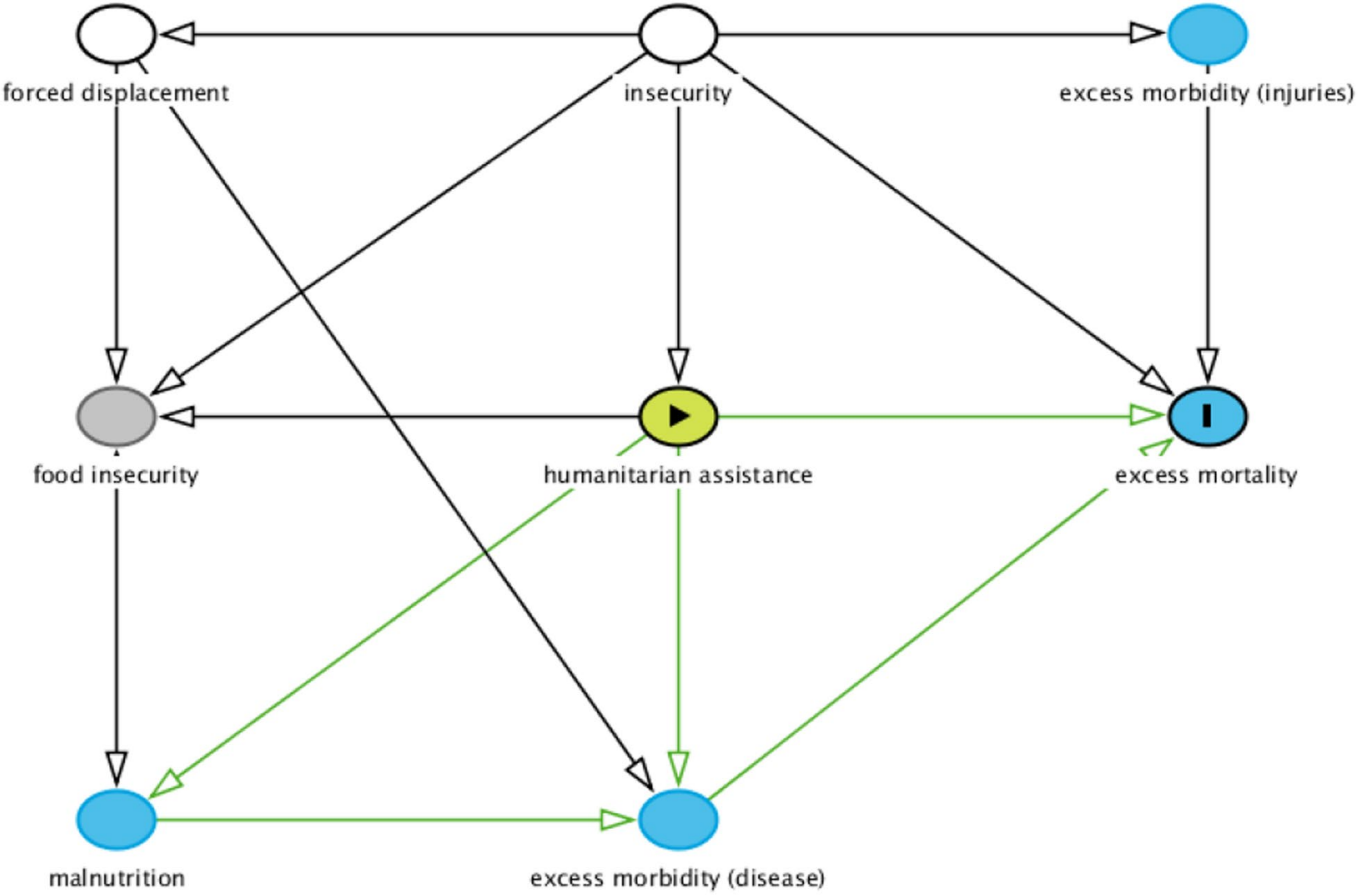
Simplified directed acyclic graph of the causal association between humanitarian assistance and excess mortality. Created with Dagitty [42]

The simplified DAG example in Fig. 4, which might apply to, say, the ongoing Tigray crisis, shows how the overall effect of HA on excess deaths (which passes through several mediating variables, e.g. malnutrition) would need to be adjusted for both insecurity and forced displacement. It may be objected that the Figure does not portray obvious feedback loops (e.g. increasing food insecurity may lead to intensified HA): this may be resolved by noting that DAGs represent causal effects in the time dimension (i.e. the state of HA today, whatever its precursor factors, has a presumed effect on mortality tomorrow).

#### Quantifying the exposure

A separate problem is how to quantify HA, i.e. the ‘exposure’ in epidemiological parlance. As outlined under question 2, HA may be parameterised in terms of its appropriateness and performance, but these quantities are difficult to combine into a single metric. Moreover, HA is the summation of multiple services across several sectors (e.g. provision of clean water; cash transfers; cholera vaccination), each of which has variable performance as captured by availability, coverage and, for some services, quality (see above). Decomposing HA into these multiple exposures is not statistically intractable, but has four disadvantages: (i) interpreting results becomes difficult if one has to simultaneously consider a large number of effects; (ii) the more associations one investigates, the higher the chance of type I error (concluding that there is an association when there in fact isn’t); (iii) because the sub-effects are likely to be small, sample sizes required to observe them with sufficient statistical confidence would be prohibitive (i.e. high type II error risk); and (iv) some of the exposures will mediate (or be on the causal pathway of) others (e.g. the effect of food security services will be mediated by nutrition services), which creates inferential complexity or, worse, may mask some of the effects.

A potential solution would be to create (globally applicable) semi-quantitative composite scores (perhaps summating appropriateness and performance separately) from information across all sectors. This task may be facilitated by identifying ‘signal’ functions of an appropriate and/or performant humanitarian response for each sector: for example, instead of collecting data on each individual humanitarian health service (a potentially monumental task), one could summarise the health sector’s response by capturing data on a shortlist of key operational indicators, such as outpatient utilisation rate, inpatient case-fatality ratio, measles vaccination coverage, antenatal visit coverage, etc. These indicators should be selected on the basis that they can feasibly be measured and, critically, that they correlate well with overall humanitarian appropriateness or performance across the sector they represent: these correlations should, in turn, be validated empirically before constructing any composite scores. Useful examples include the standardised indices measured by the WHO/UNICEF Joint Monitoring Programme for Water Supply, Sanitation and Hygiene [43], and balanced scorecards developed for health service evaluation [44–46] and health security [47].

#### Quasi-experimental approaches

What methods can concretely be leveraged to answer question 3? A number of so-called ‘quasi-experimental’ approaches [48] (see also https://www.betterevaluation.org/en/approaches) are available for evaluating real-life interventions in situations where randomised trials are unfeasible or unethical. Among these, at least three, all involving extensive statistical analysis, may have some utility to answer our question.

Statistical models of association can be used to quantify the effect size: these would combine either survey or surveillance estimates of mortality with exposure and potential confounder data, and estimate the relative rate of mortality for different HA levels. This approach bears the limitations of ecological analysis, i.e. causation is hard to establish, although a dose–response effect of HA and exploration of suitable time lags between HA and mortality, combined with careful adjustment for confounding, may strengthen causal inference.

Interrupted time series [49] is an approach that analyses changes in the trend and slope of the outcome (mortality) before/after the exposure (e.g. an intervention, in this case HA) is introduced: this approach generally considers the entire geographical area of interest, and can thus accommodate data that are not spatially granular (e.g. at crisis level), but requires frequent and consistent measurement (e.g. weekly, monthly) of the outcome. It also implies a fairly long period of data collection before HA is introduced, and thus might only be applicable in rare situations where HA has been introduced considerably late in the crisis timeline, but mortality has been measured consistently even before this, e.g. through vital event registration or prospective surveillance. The method may more readily help quantify the effect of *relative* changes in HA intensity or the introduction of specific humanitarian services, for example in refugee camps where mortality surveillance is common.

A potentially more applicable approach is propensity-score matching [50]: this would consist of identifying analysis units (e.g. district-months) that are similar to each other in various characteristics (e.g. demography, baseline disease burden, conflict intensity) *not* themselves dependent on HA, but differ by the presence or intensity of HA. The method proceeds in four steps: (i) estimate each unit’s propensity score (constructed from all available matching variables), (ii) select a matching algorithm (e.g. among units with a similar propensity score, those with ‘high’ levels of HA are matched with the nearest units with ‘low’ HA levels, etc.); (iii) check that exposure and control groups do indeed have similar characteristics; and (iv) estimate the effect of HA as the mean difference in mortality between matched exposure and control units. This method effectively attempts to partition the region and period of analysis so as to approximate the intervention and control groups that one would be able to form through random allocation, thereby facilitating causal inference. Accordingly, data used for matching should be sufficiently wide-ranging and variable to yield ‘true’ matches (i.e. exhaustively capture factors associated with mortality, other than HA) [51]. Moreover, depending on how HA is allocated in reality, it is possible that no amount of matching would remove differences between units that receive high versus low levels of HA.

Any of the above methods, if locally feasible, should yield an estimate of the association between HA and risk of mortality (for example, a rate ratio of 0.75 comparing the group exposed to HA to that unexposed would signify that those exposed had three-fourths the risk of dying as those unexposed). Such measures of association should be readily convertible into more meaningful estimates of the ‘effectiveness’ of HA (e.g. 25% reduction in mortality in the above example), and, if population denominators are well-established, the absolute number of deaths averted – a potentially compelling piece of evidence for advocacy and resource mobilisation. A common limitation would be that the effect estimated would be on total, not excess mortality.

The complexity of the above options should not be understated. Firstly, data requirements are likely to be substantial: these methods generally require a large number of ‘data points’, i.e. independent estimates of death rate for different locations and periods within the crisis person-time (and, for interrupted time series, the pre-crisis period): this could constitute the equivalent of dozens of SMART surveys, or multiple sequential monthly observations from different sites under demographic surveillance. We have previously fitted statistical models of crisis-attributable mortality with as few as 74 surveys (Somalia [10]), but these models were for prediction, not inference on a specific effect size. For each such outcome data point, analysts would also need to collect a wealth of corresponding data on the HA exposure and potential confounders (see above), or, in the case of propensity-score matching, a set of variables on conditions other than HA that different analysis units within the crisis person-time can be matched on. Such data must also be of sufficient quality to avoid regression dilution [52] or random misclassification bias, namely underestimation of the effect size due to noise in the dataset. In our group’s experience of undertaking small-area estimation predictive modelling of crisis-attributable mortality in South Sudan, Somalia, Nigeria and the Democratic Republic of Congo, routinely collected humanitarian datasets are often fragmentary, unstandardised and require extensive curation, or are simply unusable. Therefore, undertaking a quasi-experimental evaluation of HA’s mortality impact along the lines described here would probably entail prospective, well-supervised data collection supported by a sufficient evaluation budget and team. Moreover, such analyses all require in-depth expertise in data science and statistics.

#### A social autopsy option?

Instead of mortality averted, one could attempt to measure its complement—specifically, the proportion of deaths *not* averted by HA (with 0% as the target). This amended question may be amenable to individual- rather than population-level analysis: specifically, if a sufficiently large number of deaths are identified through a representative method (e.g. a retrospective survey, a key informant study or prospective surveillance), a combination of verbal autopsy [53] (questionnaires that seek to establish the probable cause of death, as related by next-of-kin) and social autopsy [54] (questionnaires that explore the circumstances leading up to the death) instruments could be used to specifically investigate which of these could have reasonably been averted by humanitarian services. For example, the death of a child from acute malnutrition could be classified as avertable by HA if it resulted from a worsening of livelihoods due to the crisis, the household did not receive sufficient food security support and/or the child did not benefit from timely initiation of nutritional therapy. In a less straightforward example, an adult death due to chronic kidney disease (CKD) might be ascribed to insufficient HA if clinical management of CKD was available pre-crisis (e.g. in Syria), but the adult did not receive this during the crisis period; if the region did not have pre-existing CKD care (e.g. South Sudan), the death might not be considered within the remit of HA. A death due to shelling of civilian housing might be considered unavertable by HA if the individual died immediately, but possibly avertable if the individual’s wounds were insufficiently managed. In the same example, one might need to account for whether prevailing security conditions would have realistically allowed for ambulance transport or functional trauma surgery.

The above examples point to some likely limitations of the social autopsy approach: lengthy, detailed questionnaires, and uncertainty in many cases as to how the death should be tallied within the proportion being estimated. Moreover, while this approach would capture the consequences of inadequate humanitarian food security and public health services, it would probably overlook other humanitarian sectors, such as shelter, protection and education, which also contribute, albeit more distally, to human survival. While verbal autopsy is fairly established, substantial work would be required to develop and validate context-adaptable social autopsy questionnaires and processes for classifying deaths. On the other hand, the approach also carries potential advantages: estimating a proportion with accuracy sufficient for action would require moderate sample sizes; verbal and social autopsies would be integrated within data collection (e.g. surveys) to estimate the actual death rate; the method would give some voice to affected people; and the information generated could be highly actionable, i.e. point to specific barriers in accessing services that could be removed, or health problems that are insufficiently addressed.

## Discussion

### Evaluative packages

This paper has tried to identify potential approaches to illuminate what may arguably be the most critical question for evaluating humanitarian responses. The options outlined above need not be considered mutually exclusive: rather, it may be helpful to conceive of ‘packages’ of methods that can be applied during different phases. For example, it may be helpful to accompany the implementation of a response with methods to answer questions 1 and 2 above, i.e. ongoing monitoring of death rates, combined with audits of the appropriateness and performance of humanitarian services. A study of mortality reduction attributable to HA (question 3) may be conducted during periodic or post-crisis evaluations, though, as discussed, the quasi-experimental options outlined would likely require preparatory data collection throughout the response. Combining questions 1 and 3 could also be attempted in real-time if social autopsies are integrated with ongoing death rate estimation.

While this paper set out to identify solutions, it is imperative that the above or other options are subjected to extensive methodological research and testing in different scenarios, in what would surely amount to a multi-year, multidisciplinary global programme of work, ideally led by or at least extensively involving academics and evaluators from countries affected by crises. Such research need not all take place in advance of any attempt at actual measurement: rather, a careful ‘learning by doing’ approach may be preferable, so long as analysts and end-users faithfully represent potential bias arising from methods under development.

### It’s all too hard and expensive. Or is it?

The challenges and requirements set out above for each method explored may leave HA stakeholders despondent. Systematic measurement of HA’s mortality impact will undoubtedly require an unprecedented commitment of resources and, furthermore, predictable governance arrangements to ensure that measurement occurs even in the most politically complex crises. For transparency reasons, the task may need to be attributed to a specialised auditing unit. While these steps may seem extraordinary, what may arguably be considered more unusual is the near-lack of evidence on the extent to which HA achieves one of its existential aims. Against a yearly financial outlay of 27–31 billion USD since 2016 [55], earmarking a fraction of humanitarian funding for mortality evaluation may be viewed as a minimal investment in governance and accountability—one that, for example, public health services in high-income countries routinely make to produce publicly available statistics of performance and, critically, identify instances of low performance or medical malpractice that warrant remedial action. Indeed, the cost of measurement needs to be weighed against the likely efficiencies that would result from more evidence-based, appropriate and equitable humanitarian responses. Measurement and evaluation are, at their best, a cost-saving measure.

### Prevention versus response

Lastly, some crises can be prevented, e.g. through reconciliation and negotiated solutions to conflicts; many can be mitigated to some extent if affected governments, civil society actors and people themselves are empowered to build resilience against threats such as drought, sudden natural disasters and disruptions to public services. The farther upstream these measures intervene in the pathway to crisis-attributable mortality, the harder it becomes to specify counterfactuals (i.e. what might have happened in their absence) or control groups, and thus quantify their impact. A justifiable focus on measuring the true effect of ‘reactive’ HA should thus not obscure the potentially greater value and efficiency of such preventive actions.

## Data Availability

Not applicable.
